# Proteomic detection of a large amount of SCGFα in the stroma of GISTs after imatinib therapy

**DOI:** 10.1186/1479-5876-9-158

**Published:** 2011-09-23

**Authors:** Luca Da Riva, Fabio Bozzi, Piera Mondellini, Francesca Miccichè, Elena Fumagalli, Elena Vaghi, Eva Tarantino, Veronica Huber, Alessandro Gronchi, Elena Tamborini, Marco A Pierotti, Silvana Pilotti, Italia Bongarzone

**Affiliations:** 1 Proteomics Laboratory, Department of Experimental Oncology and Molecular Medicine, Fondazione IRCCS Istituto Nazionale dei Tumori, Milan, Italy; 2 Experimental Molecular Pathology, Department of Pathology, Fondazione IRCCS Istituto Nazionale dei Tumori, Milan, Italy; 3 Department of Cancer Medicine, Fondazione IRCCS Istituto Nazionale dei Tumori, Milan, Italy; 4 Unit of Immunotherapy of Human Tumors, Department of Experimental Oncology Fondazione IRCCS Istituto Nazionale dei Tumori, Milan, Italy; 5 Department of Surgery, Fondazione IRCCS Istituto Nazionale dei Tumori, Milan, Italy; 6 Scientific Directorate, Fondazione IRCCS Istituto Nazionale dei Tumori, Milan, Italy

## Abstract

**Background:**

Gastrointestinal stromal tumors (GISTs) are the most frequent mesenchymal tumors to develop in the digestive tract. These tumors are highly resistant to conventional chemotherapy and only the introduction of imatinib mesylate has improved the prognosis of patients. However, Response Evaluation Criteria in Solid Tumors are inappropriate for assessing tumor response, and the histological/pathological response to imatinib is variable, heterogeneous, and does not associate with clinical response. The effects of imatinib on responding GISTs are still being explored, and few studies correlate the clinical response with the histological response after pharmacological treatment. Recently, apoptosis and autophagy were suggested as possible alternative mechanisms of pharmacological response.

**Methods:**

Here, we used a proteomic approach, combined with other analyses, to identify some molecular stromal components related to the response/behavior of resected, high-risk GISTs after neoadiuvant imatinib therapy.

**Results:**

Our proteomic results indicate an elevated concentration of Stem Cell Growth Factor (SCGF), a hematopoietic growth factor having a role in the development of erythroid and myeloid progenitors, in imatinib-responsive tumor areas. SCGFα expression was detected by mass spectrometry, immunohistochemistry and/or western blot and attributed to acellular matrix of areas scored negative for KIT (CD117). RT-PCR results indicated that GIST samples did not express SCGF transcripts. The recently reported demonstration by Gundacker et al. [1] of the secretion of SCGF in mature pro-inflammatory dendritic cells would indicate a potential importance of SCGF in tissue inflammatory response. Accordingly, inflammatory infiltrates were detected in imatinib-affected areas and the CD68-positivity of the SCGF-positive and KIT-negative areas suggested previous infiltration of monocytes/macrophages into these regions. Thus, chronic inflammation subsequent to imatinib treatment may determine monocyte/macrophage recruitment in imatinib-damaged areas; these areas also feature prominent tumor-cell loss that is replaced by dense hyalinization and fibrosis.

**Conclusions:**

Our studies highlight a possible role of SCGFα in imatinib-induced changes of GIST structure, consistent with a therapeutic response.

## Background

Gastrointestinal stromal tumors (GISTs) are the most frequent mesenchymal tumors to develop in the digestive tract. They typically arise from the stomach (40-70%) or small intestine (20-40%) but can also occur in the colon-rectum (10-15%) and rarely in the esophagus. At least 10-30% of tumors are discovered incidentally during laparotomy, endoscopy, or other imaging studies; 15-47% of patients present with overt metastatic disease and common sites of metastases include liver, peritoneum, and omentum [2-4]. GISTs are thought to originate from the interstitial cells of Cajal or their precursor cells [5]. Most GISTs are characterized by gain-of-function mutations in the genes encoding *KIT *and platelet-derived growth factor receptor alpha (*PDGFRA*) [6], mutations that appear to be mutually exclusive. The emerging role of stem cell factor (SCF) as the ligand of the receptor tyrosine kinase KIT [7-9] suggests that an autocrine-paracrine loop serves as a possible further mechanism of action [10]. However, the SCF/KIT system plays an important role not only in the differentiation and proliferation of interstitial cells of Cajal and the development of GISTs but also in the development of hematopoietic cells such as mast cells, erythroblasts, and melanocytes [11].

GISTs are highly resistant to conventional chemotherapy [12]; in the past decade, the introduction of imatinib mesylate (Gleevec^®^, Novartis Pharmaceutical Corporation, NJ, USA), a KIT receptor blocker, has significantly improved the prognosis of GIST patients. Tumor response depends on the presence/absence and type of mutations in KIT or PDGFR. Unfortunately, the major problem with imatinib treatment is resistance, mainly secondary resistance that generally evolves in most patients after a median of two years of therapy [13,14].

Eighty to eighty-five percent of patients with advanced GISTs exhibit an initial benefit from imatinib treatment; however, the response level varies from rapid and gross reduction in tumor volume to little or no tumor shrinkage (described as stable disease) [6]. Size-based response criteria such as the World Health Organization criteria or the current international Response Evaluation Criteria in Solid Tumors are thus thought to underestimate the response and are not appropriate tools to assess tumor response to imatinib [15]. Consequently, the clinical management of these patients and the criteria used to assess clinical response to imatinib therapy have recently been redefined [16]. In accordance with previously reported criteria, GISTs can be classified using the following scores: high responders, 0 to <50% residual viable tumor cells with no mitosis, and no obvious Ki-67 immunostaining; moderate responders, >50% to 90% tumor cells, no mitosis, and Ki-67 immunostaining in 0 to <10% of cells; low responders, >50% to 90% tumor cells, mitotic index > 10/50 high-power fields, Ki-67 immunostaining in 20-30% or >30% of cells; and non-responders, >90% tumor cells [17,18].

The histological/pathological response of GIST to imatinib therapy is also variable and heterogeneous from nodule to nodule within the same resection, as well as within the same lesion, and does not correlate well with clinical response. Limited studies of the histopathological changes in imatinib-treated patients indicate a significant change in the appearance of the tumor tissue following preoperative systemic imatinib therapy in GISTs; alterations particularly occur in the stromal compartment and are visible during standard pathological assessment. The density of the tumor and the number of intratumoral vessels decrease significantly and areas of cystification and hemorrhage are clearly visible. Tumors become homogeneous and hypodense. However, even in highly responsive tumors, microscopic foci of viable cells are seen either as isolated tumor cells or as distinct micronodules embedded in an extensively hyalinized background.

The molecular effects of imatinib on responding GISTs are currently being explored. Apoptosis (programmed cell death type I) has been frequently described in GIST cell lines treated with imatinib mesylate [19,20]; importantly, McAuliffe et al. [21] indicate that the action of imatinib may be both cytotoxic (by evidence of apoptosis) and cytostatic. Recently, autophagy (programmed cell death type II) has been suggested as a possible alternative mechanism of response to the imatinib mesylate treatment in clinical biopsies [22]. Autophagy is the major self-degradative process in eukaryotic cells and has multiple physiological functions, including protein degradation, organelle turnover, and response of cancer cells to chemotherapy [23].

In this report, we used a proteomic approach in combination with other analyses to investigate the protein composition of highly responsive, resected GISTs after imatinib mesylated neoadjuvant therapy. Our aim was to identify several molecular components of the stroma with expression patterns possibly related to tumor response/behavior.

## Methods

### Patients and materials

GIST diagnosis was previously performed according to currently accepted criteria and confirmed by molecular analysis demonstrating activating mutations in the KIT receptor [24]. Tumor characteristics and treatment are detailed in Table 1. A tumor tissue sample (GIST 1) from an untreated patient was included in the analysis. Specimens from each patient, verified by histopathology, were snap-frozen in liquid nitrogen and stored in cryotubes in liquid nitrogen. The specimens (one sample per case) were assessed for response to imatinib mesylate treatment based on the morphological criterion of residual cellularity. Written informed consent to participate in the study was obtained from each patient before surgery in accordance with the ethical guidelines of our institute. Plasma samples were collected under a protocol approved by our institutional review board and the donors provided written informed consent.

**Table 1:** Clinical, pathological and molecular characterists of GISTs

Case	Anatomical location	Molecular Status	Clinical response^1^	Pathological response^2^	Tumor Sample	Size^3 ^(cm)
GIST 1	Stomach	KIT exon 11 Del 558-563	Non treated	Non treated	Primary	>10

GIST 2	Ileum	KIT exon 11 L576P	Stable disease	>90% of viable cells-non responder	Recurrence	2-5

GIST 3	Esophagus	KIT exon 11 Del 554-558	In response	10% of viable cells-high responder	Primary	5-10

GIST 4	Stomach	KIT exon 11 Del 557-558	In response	10% of viable cells-high responder	Primary	>10

GIST 5	Stomach	KIT exon 11 V559D	In response	10% of viable cells-high responder	Primary	>10

GIST6	Stomach	PDGRF alpha D842V	Unresponsive	25% of viable cells-high responder	Recurrence	< 2

GIST7	Ileum	KIT exon 9 Dup 502-503	Unresponsive	>90% of viable cells-non responder	Liver metastasis	5-10

GIST8	Pelvis	KIT exon 11 Del 558-560 and V557C	Unresponsive	>5% of viable cells-high responder	Liver metastasis	2-5

GIST9	Stomach	PDGFR alpha D842V	Unresponsive	>90% of viable cells-non responder	Primary	2-5

GIST10	Stomach	KIT wt	Unresponsive	>90% of viable cells-non responder	Primary	>10

GIST11	Stomach	KIT Dup P577-K581	Unresponsive	>90% of viable cells-non responder	Primary	5-10

GIST12	Omentum	KIT Dup A502-Y503 and N822K	Unresponsive	>90% of viable cells-non responder	Metastasis	< 2

GIST13	Intestin	KIT K642E and N822K	Unresponsive	>90% of viable cells-non responder	Primary	>10

GIST14	Intestin	KIT Del M552-Y553 and E554K	In response	50-90% of viable cells-moderate responder	Recurrence	5-10

GIST15	Stomach	KIT K642E	In response	50-90% of viable cells-moderate responder	Metastasis	5-10

GIST16	Intestin	KIT Dup 502-503	In response	50-90% of viable cells-moderate responder	Primary	< 2

^1 ^Evaluated on the frozen material used for the proteomic/biochemical analyses [16].

^2 ^According to refs 17 and 18.

^3 ^The size of the tumor was measured considering the maximum diameter.

### Protein extraction

Tumor specimens were pulverized in a Mikro-Dismembrator II (B. Braun Biotech International, Melsungen, Germany). The pulverized tissue samples and the cell pellets from cell culture were recovered in ice-cold buffer containing 50 mM HEPES (pH 7.6), 150 mM NaCl, 10% glycerol, 1% Triton X-100, 1.5 mM MgCl_2_, 1 mM EGTA, 10 mM Na_4_P_2_O_7_, 100 mM NaF, 1 mM PMSF, 1 mM sodium orthovanadate, and Complete Mini protease inhibitors cocktail (Roche, Milan, Italy) according to the manufacturer's instructions. After 30 min incubation with gentle rocking at 4°C, lysates were cleared by centrifugation for 20 min at 13,000 rpm. Supernatants were collected and protein quantification was performed with the BCA™ Protein Assay Kit (Thermo Scientific, Milan, Italy) according to the manufacturer's instructions.

### One-dimensional SDS-PAGE

Thirty micrograms of total extract from GIST samples and of plasma were loaded on one-dimensional 4-12% NuPAGE^® ^precast gels (Invitrogen, Milan, Italy). Proteins were visualized with G250 Coomassie Blue (Bio-Rad, Milan, Italy) by standard procedures.

### In-gel tryptic digestion, matrix-assisted laser desorption/ionization-time of flight-mass spectrometry (MALDI-TOF-MS), and peptide mass fingerprinting

For protein profiling, protein bands were excised from Coomassie-stained preparative gels and processed as previously described [25]. MALDI-TOF-MS was carried out using a Voyager-DE STR (Applied Biosystems, Milan, Italy), equipped with a nitrogen laser (337 nm).

Monoisotopic peptide masses were analyzed using the Aldente software http://www.expasy.org/tools/. Input was searched according to: Aldente, UniProtKB/SwissProt; predefined taxon, Mammalia; Spectrometer internal error max, 25. Only proteins identified in at least three separate experiments were considered.

### OFFGEL protein fractionation

A preparative-scale OFFGEL was used for isoelectric focusing of proteins. To perform protein fractionation according to isoelectric point, the 3100 OFFGEL Fractionator and the OFFGEL Kit 3-10 (Agilent Technologies, Milan, Italy) were used following the manufacturer's instructions [26]. The device was set up for the 24-fraction separation using the 24 cm-long IPG gel strip with a linear pH gradient from 3 to 10. The proteins were separated in a two-phase system consisting of a liquid upper phase (focusing buffer provided by the supplier) separated in wells and a lower IPG gel strip phase. The sample was focused using the recommended method for 24-well OFFGEL fractionation with a maximum current of 50 μA. The separation method consisted of a cooling platform temperature of 15°C with electrical setting parameters of 8,000 V/h, 100,000 V, 200 W, and 50 μA/strip. The focusing was stopped after the total voltage reached 64 kVh. After focusing, samples were recovered from each well and transferred to individual microtubes. Corresponding protein fractions were purified with the 2-D Clean Up (GE Healthcare, Milan, Italy) and the protein pellets were dissolved in running sample buffer compatible with one-dimensional SDS-PAGE. For protein profiling, protein bands were excised from Coomassie-stained preparative gels and processed as previously described [25].

### Cell culture

The human papillary thyroid carcinoma cell line, TPC1, was grown adherently in Dulbecco's Modified Eagle's Medium (Gibco, Milan, Italy) supplemented with 10% fetal bovine serum HyClone (Celbio, Milan, Italy) and 1 mM sodium pyruvate (Lonza, Milan, Italy). Human Embryonic Kidney (HEK) 293T (ATCC number CRL-1573) cells were grown adherently in Dulbecco's Modified Eagle's Medium supplemented with 10% fetal bovine serum and L-glutamine (Lonza).

### Conditioned medium concentration

An equal volume (~5 ml) of conditioned medium for each sample (mock and transfected cells) was loaded into a spin concentrator (Agilent Technologies) with a 5 kDa molecular weight cut-off and centrifuged at 4,000 rpm and 10°C until samples were concentrated to a final volume of 150-200 μl. Protein concentration was determined by BCA assay.

### Western blot

An equal amount of protein for each sample (30 μg) was loaded on a one-dimensional 4-12% NuPAGE^® ^precast gel (Invitrogen). Proteins were transferred in NuPAGE^® ^transfer buffer (Invitrogen) and 20% ethanol onto a nitrocellulose membrane (Hybond™-C Super, Amersham Biosciences, Milan, Italy) and checked for equal sample loading by Red Ponceau S (Sigma-Aldrich, Milan, Italy) staining. Blots were blocked for 1 h with TBS (10 mM Tris-HCl [pH 7.5], 150 mM NaCl) plus 0.1% Tween 20 (TBS-T buffer) containing 1% bovine serum albumin (Sigma-Aldrich) and 3% ovalbumin (Sigma-Aldrich), then hybridized in the same buffer with specific antibodies at 4°C overnight using the recommended dilutions. After incubation, the blots were washed in TBS-T buffer and incubated for 1 h at room temperature in previously described buffer using appropriate secondary antibodies (1:4,000). After incubation, the blots were washed in TBS-T buffer, and immunoreactive proteins were visualized using an enhanced ECL system (ECL^® ^Western blotting detection reagents, GE Healthcare Life Sciences, Milan, Italy) according to the manufacturer's protocol. Monoclonal mouse anti-human SCGF/CLEC11a antibody was supplied by R&D Systems (Milan, Italy). The ECL^® ^anti-mouse IgG, horseradish peroxidase-linked whole antibody from sheep was obtained from GE Healthcare Life Sciences.

### Immunohistochemistry

Immunohistochemistry was performed on 2-μm formalin-fixed and paraffin-embedded sections of representative tumoral areas deparaffined in xylene and rehydrated in graded alcohols. Endogenous peroxidase activity was blocked by treatment for 10 min with 0.3% hydrogen peroxide in distilled water. Antigen retrieval was obtained by autoclaving for 15 min.

The slides were cooled under tap water, washed three times in 0.05 M phosphate-buffered saline plus 0.1% Triton, and incubated with ultra v-block (Lab Vision Corp, Newmarker, UK) for 10 min at room temperature. Then, they were incubated at room temperature for 1 h with the monoclonal mouse anti-human SCGF/CLEC11a antibody (R&D Systems) diluted 1:400 in citrate buffer (pH 6). The slides were washed again three times in 0.05 M phosphate-buffered saline plus 0.1% Triton and developed using the Ultra Vision LP Volume Detection System (Lab Vision Corp). After washes in 0.05 M phosphate-buffered saline, peroxidase activity was detected with diaminobenzidine for 10 min in the dark. The slides were counterstained with hematoxylin. Immunohistochemical analysis for KIT was performed using an antibody against CD117, as previously described [27]. Positivity was defined as the detection of immunopositivity in >90% of cells.

### Plasmid construction

Total RNA of the TPC1 cell line was prepared using a TRIZOL reagent (Life Technologies, Italy), the oligo(dT)-primed cDNA was synthesized using a RT-PCR kit (Stratagene, Milan, Italy). Oligonucleotides 5'-CCAAGCTTTCCAGCTTAATGCAG-3'(forward) and 5'-TAAAGCGGCCGCCCCGCTAGAA-3'(reverse) were used in PCR to amplify the full human SCGF- α (hSCGF-α, UniProt accession number Q9Y240) sequence. Taq Phusion^® ^High Fidelity DNA polymerase (Finnzymes, Espoo, Finland) was used with the following thermal cycling conditions: initial denaturation at 98°C for 30 sec; 35 cycles of denaturation at 98°C for 10 sec, annealing at 60°C for 30 sec, extension at 72°C for 40 sec; and final extension at 72°C for 7 min. The hSCGF-α cDNA was subcloned into the pcDNA3.1 plasmid vector (Invitrogen). The plasmid expressing hSCGF-β was obtained by mutagenesis of the plasmid expressing hSCGF-α using the Quick-Change^® ^II XL Site-Directed Mutagenesis Kit (Stratagene, Milan, Italy), according to the manufacturer's protocol. The DNA sequences contained in both vectors were checked by automatic sequencing.

### Cell transfection

HEK 293T cells were transiently transfected by calcium phosphate precipitation, as previously described [28], with the pcDNA3 expression vector (Invitrogen) alone (mock) or with the plasmid carrying the insert for SCGF-β.

### Removal of O-linked oligosaccharides from SCGF

Suitable amounts (based on western blotting with anti-SCGF) of cell and tissue protein extracts and secreted proteins were incubated at 37°C for 4 h with O-glycosidase (Sigma-Aldrich) and α(2→3,6,8,9)neuraminidase (sialidase, Sigma-Aldrich) according to the manufacturer's protocol. The digested samples were analyzed by western blotting with anti-SCGF.

## Results

**Figure 1:**
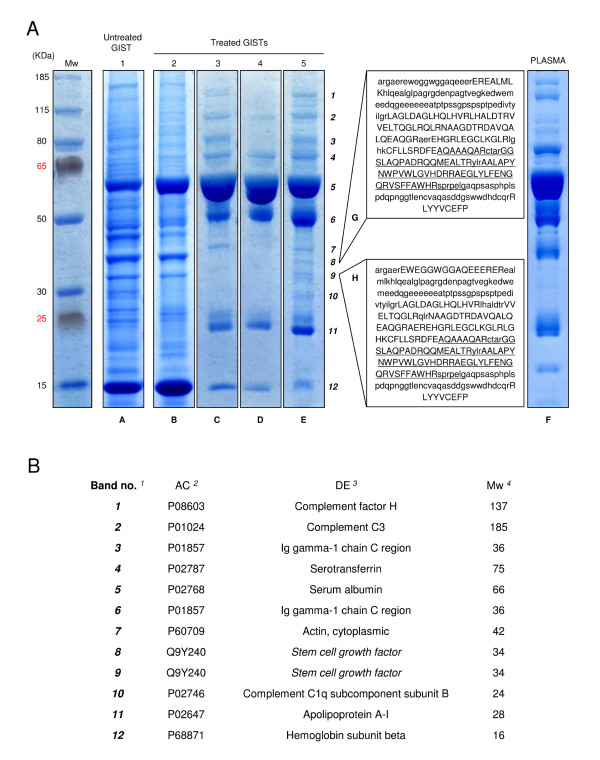
**Protein expression in GISTs from patients treated with imatinib mesylate**. **A) **SDS-PAGE (4-12% bis-Tris gel) of total protein extracts from three highly responsive GIST patients (lanes C-E) and from one poorly responsive patient with stable disease (lane B). A tumor tissue sample from an untreated GIST patient is shown as the control (lane A). A pool of plasma samples from healthy subjects (lane F) is shown for comparison with GIST extracts. Lanes G and H demonstrate the protein coverage of SCGF isoforms assessed by MALDI-TOF-MS of tryptic peptides obtained from digestion of the proteins in bands 8 and 9, respectively. The boxed sequences correspond to the sequence without signal peptide (amino acids 1-21) of SCGF-α (Q9Y240) that is available in the Aldente (ExPASy Proteomics Server) database. Capital letters indicate the sequence detected by MS, while the underlined sequence indicates the 78 amino acids that are lacking in the β-form. Bold numbers denote the proteins identified by MS and listed in panel B. **B**) Proteins identified in GIST 5 (lane E) after SDS-PAGE fractionation, tryptic digest, and MALDI-TOF-MS. ^1^Number refers to the specific band position on the SDS-PAGE in Figure 1A, lane E. ^2^Swiss-Prot/TrEMBL accession line. ^3^Swiss-Prot/TrEMBL description line. ^4^Theoretical protein mass.

To explore the protein expression pattern of GIST samples treated with imatinib mesylate, protein extracts from five GISTs were loaded onto one-dimensional SDS-PAGE and visualized by Coomassie staining. These five samples consisted of one untreated tumor (GIST 1), three tumors from high-responder patients (GISTs 3-5), and one tumor (GIST 2) from a patient with clinically stable disease but who was scored as a non-responder (Table 1). The protein patterns of the highly responsive tumors (Figure 1, lanes C-E) were uniform and contained fewer protein bands than the other samples (Figure 1, lanes A and B). The staining patterns of the GIST samples did not differ consistently from that of a pool of plasma samples from healthy donors (Figure 1, lane F). MS of gel bands obtained after OFFGEL fractionation of the extracts (data not shown) confirmed that the majority of proteins were blood components (Table 2).

**Table 2:** List of proteins identified after OFFGEL and SDS-PAGE fractionation, tryptic digest, and MALDI-TOF MS analysis

No.	AC*^1^*	DE*^2^*	Mw*^3^*	pI*^4^*	Cov*^5^*
1	P01009	Alpha-1-antitrypsin	44	5.4	47

2	P04217	Alpha-1B-glycoprotein	52	5.6	38

3	P02750	Leucine-rich alpha-2-glycoprotein	34	5.7	22

4	P01023	Alpha-2-macroglobulin	161	5.9	28

5	P01011	Alpha-1-antichymotrypsin	45	5.3	32

6	P60709	Actin, cytoplasmic 1	42	5.3	65

7	P63261	Actin, cytoplasmic 2	42	5.3	65

8	P43652	Afamin	67	5.6	42

9	P02768	Serum albumin	66	5.7	66

10	P01008	Antithrombin-III	49	6.0	30

11	P08758	Annexin A5	36	4.9	69

12	P02647	Apolipoprotein A-I	28	5.3	74

13	P06727	Apolipoprotein A-IV	43	5.2	43

14	P00450	Ceruloplasmin	120	5.4	15

15	P00751	Complement factor B	83	6.7	36

16	P08603	Complement factor H	137	6.1	46

17	P01024	Complement C3	185	6.0	11

18	P0C0L4	Complement C4b-A	84	5.3	21

19	P0C0L5	Complement C4b-B	84	5.4	21

20	P02765	Alpha-2-HS-glycoprotein chain A	30	4.5	32

21	P02679	Fibrinogen gamma chain	48	5.2	33

22	P02792	Ferritin light chain	20	5.5	45

23	P68871	Hemoglobin subunit beta	16	6.8	84

24	P00738	Haptoglobin	43	6.1	40

25	P08238	Heat shock protein HSP 90-beta	83	5.0	24

26	P07900	Heat shock protein HSP 90-alpha	85	4.9	18

27	P01876	Ig alpha-1 chain C region	38	6.1	29

28	P01857	Ig gamma-1 chain C region	36	8.5	28

29	P01859	Ig gamma-2 chain C region	36	7.7	26

30	P19827	Inter-alpha-trypsin inhibitor heavy chain	71	6.3	17

31	P13645	Keratin, type I cytoskeletal 10	60	5.1	24

32	P35527	Keratin, type I cytoskeletal 9	62	5.2	16

33	P04264	Keratin, type II cytoskeletal 1	66	8.2	26

34	P01042	Kininogen-1	70	6.2	22

35	P00734	Prothrombin	65	5.2	50

36	P02787	Serotransferrin	75	6.7	47

37	P02766	Transthyretin	14	5.4	80

38	P02774	Vitamin D-binding protein	51	5.2	53

39	P25311	Zinc-alpha-2-glycoprotein	32	5.6	22

*^1 ^*Swiss-Prot/TrEMBL accession line.

*^2 ^*Swiss-Prot/TrEMBL description line.

*^3 ^*Theoretical protein mass.

*^4 ^*Theoretical protein isoelectric point.

*^5 ^*Number of amino acids present in at least one peptide over the number of amino acids of the protein.

In the GIST 5 extract, Coomassie staining revealed bands (bands 8-10 in Figure 1A, lane E, and Figure 1B) that were not present in the plasma sample. Bands 8 and 9 corresponded to SCGF, also known as C-type lectin domain family member 11A (CLEC11A), an important hematopoietic growth factor with burst-promoting activity for human bone marrow erythroid progenitors [29]. Band 10 corresponded to C1q, the initiator of the classical complement cascade [30].

**Figure 2:**
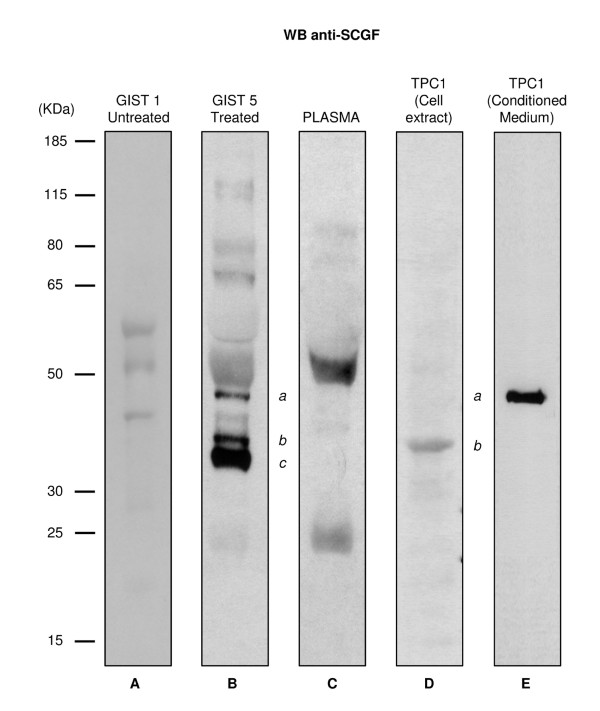
**Western blotting of SCGF expression**. Western blotting with anti SCGF-α/-β antibody of tumor tissue samples from the untreated GIST 1 patient (lane A), the SCGF-positive GIST 5 patient (lane B), a pool of plasma samples from healthy subjects (lane C), and the cell extract (lane D) and conditioned medium (lane E) from the TPC1 cell line. *(a) *indicates the mature and secreted form of SCGF-α detected in the GIST 5 sample (lane B) and in the TPC1 conditioned medium (lane E); *(b) *indicates the immature and cytoplasmatic form of SCGF-α detected in the GIST 5 sample (lane B; corresponding to band 8 in Figure 1A) and in the TPC1 cell extract (lane D); *(c) *points out the quantitatively most relevant form, exclusively present in the GIST 5 sample, corresponding to band 9 in Figure 1A.

Western blotting with anti-SCGF antibody confirmed the presence of SCGF-positive bands in GIST 5 extract (*b *and *c *in Figure 2, lane B) and highlighted the presence of a third isoform of ~46 kDa (*a *in Figure 2, lane B) that was not detected by the proteomic analysis. All three isoforms were undetectable in a plasma sample (Figure 2, lane C), consistent with the observation that the SCGF plasma concentration was too low to be analyzed by western blot. In the peripheral blood of adults, SCGF concentration can only be detected by enzyme-linked immunosorbent assay because the concentration is approximately 10-20 ng/ml [31]. Using Coomassie staining to visualize the electrophoresed SCGF proteins, we estimated at least 60 ng of protein in the GIST 5 sample. Since 30 μg of tissue extract was analyzed, we calculated the SCGF concentration to be 0.5 ng/μg in the GIST extract, which was much more protein than would be present in an equivalent amount of plasma protein (0.03-2 pg/μg).

**Figure 3:**
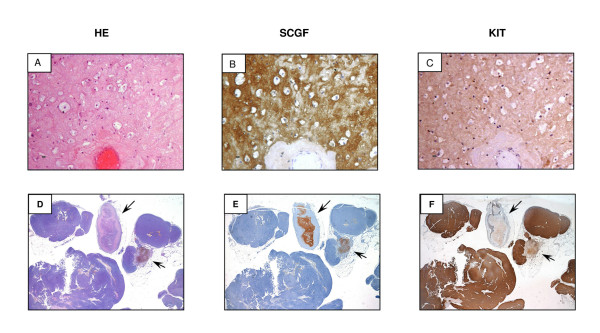
**Immunohistochemistry of SCGF expression in GIST samples**. **A) **Hematoxylin-eosin section of one representative slide from GIST 5. **B**) SCGF staining; strong SCGF positivity, consistent with western blotting, was restricted to the stroma compartment. **C**) CD117 staining; cells retained weak cytoplasmic KIT reactivity. The rare viable tumoral cells present in the responding area were SCGF-negative in the cytoplasm and the nucleus. **D**) Hematoxylin-eosin section of one representative slide from GIST 2. Highly cellulated areas indicating the absence of pathological response are visible. Only two areas showing rare tumoral cells are present. **E**) CD117 staining; all cellulated areas exhibit strong KIT expression not observed in the acellulated areas mostly composed of stroma. **F**) SCGF staining; strong SCGF expression is restricted to the non-cellulated areas, in contrast to CD117 staining.

To assess the possible origin and localization of SCGF, we performed immunohistochemistry on all five GIST samples. GIST 5 (Figure 3B) displayed a strong SCGF positivity, consistent with the western blot, that was restricted to the stroma compartment. The rare viable tumoral cells present in the responding area were negative in the cytoplasm and the nucleus. However, these cells retained weak cytoplasmic KIT reactivity (Figure 3C). The responding cases (GISTs 3 and 4) and the naïve GIST (GIST 1) were negative (Additional file 1 and data not shown). Interestingly, GIST 2 (Figure 3D-F) exhibited minimal areas of regression that were mostly depleted of tumoral cells and were SCGF-positive and KIT-negative. RT-PCR did not reveal the presence of SCGF transcripts in responding or non-responding GISTs (Additional file 2), corroborating the hypothesis that blood is likely the source of SCGF.

**Figure 4:**
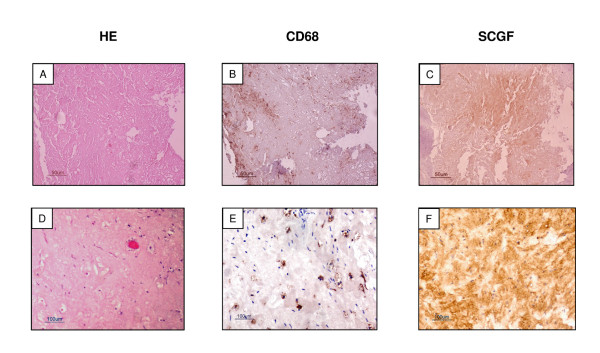
**Immunohistochemistry of CD68 and SCGF in GIST 5 and GIST 2**. **A) **Hematoxilin-eosin section of one representative slide from GIST 5. **B**) CD68 staining; CD68 positive macrophages are scattered through the section. **C**) SCGF staining; SCGF positivity was observed in the corresponding stromal area. **D**) Hematoxylin-eosin section of one representative slide from GIST 2. **E**) CD68 staining; a diffuse infiltration of CD68-positive macrophages is visible in this area. **F**) SCGF staining; SCGF positivity was observed in the corresponding stromal counterpart.

SCGF is a largely uncharacterized hematopoietic mediator that promotes enhanced erythroid progenitor formation from human bone marrow [32]. Stem cell transplantation-elevated serum SCGF levels are associated with enhanced hematopoietic recovery [32], and the differentiation of dendritic cells in mature type I inflammatory cells is accompanied by an increase in SCGF secretion [1]. Based on these observations, we hypothesized that an inflammatory reaction induced by imatinib treatment could be responsible for the SCGF positivity we observed in our GIST specimens. We therefore employed immunohistochemistry to investigate the presence of macrophage/dendritic cells using anti-CD68 antibodies in samples GIST 2 and 5. Interestingly, we observed areas of CD68 positivity in GIST 5, while GIST 2 presented a diffuse infiltration of CD68-positive macrophages. In both cases the corresponding stromal counterpart was SCGF-positive (Figure 4).

**Figure 5:**
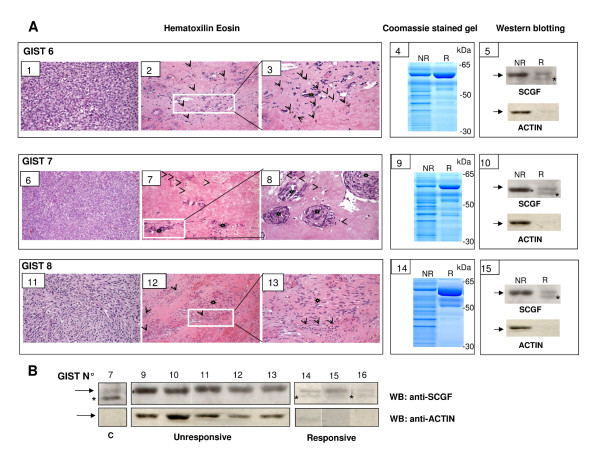
**Analysis of an independent set of imatinib-treated GIST samples**. **A) **Comparative analysis of tumoral areas from of GIST 6, 7 and 8, which resulted non-responding or responding to imatinib treatment. Panels 1-5, 6-10 and 11-15 refer to GISTs 6, 7 and 8 respectively. Panels 1, 6 and 11 represent non-responding areas; panels 2, 7 and 12 responding areas; panels 3, 8 and 13 show the inset areas of panels 2, 7, and 12. Arrows and arrowheads indicate inflammatory macrophages and lymphocytes, respectively, and asterisks residual tumor cells. Panels 4, 9 and 14 show the protein lysates prepared from adjacent tumor areas were separated onto SDS gels and stained with Coomassie-blu. Lane NR indicates the non-responding area and lane R indicates the responding area. Panels 5, 10 and 15 show NR and R protein lysates were probed with anti-SCGF and anti-actin antibodies, respectively. Note that in panels 5, 10 and 15 arrows refer to the actin band that is detected as a non-specific background band in anti-SCGF blot. As expected, due to the higher cellularity we observe a more intense actin band in NR samples than in R samples. **B**) Western blotting of SCGF expression in GIST 9-16 samples. GIST 9-13 represent protein lysates from non-responder GIST samples and GIST 14-16 protein lysates from responder samples. C corresponds to GIST 6 protein lysate. Samples were probed with both anti-SCGF and anti-actin antibodies. Arrow indicates actin band and asterisks SCGF band. In non-responder samples anti-SCGF detected exclusively the actin band.

In order to test the consistency of the relationship between SCGF expression and the histological response to imatinib, we analyzed SCGF expression in tumor areas scored as acellular or with <10% residual tumoral cells and in those areas scored as non-responsive or scarcely responsive in three imatinib-treated patients (GISTs 6, 7 and 8 in Table 1). Figure 5A summarizes the results obtained in a comparative analysis of non-affected and affected tumor areas: the hematoxylin-eosin staining of the histological sections (panels 1-3; 6-8; and 11-13); the images of the protein lysates prepared from adjacent tumor areas that were separated onto SDS gels and stained with Coomassie-blu (panels 4, 9 and 14); and, the western blotting of the same protein lysates probed by with anti-SCGF antibody (panels 5, 10 and 15). Lysates from imatinib-affected areas displayed a simplified protein band profile; notably, the anti-SCGF antibody recognized an SCGF-related band in protein lysates from responsive tumoral areas that was absent in lysates from matched non-responsive or scarcely responsive areas (Figure 5A). These observations strongly support a relationship between SCGF positivity and low cellularity due to imatinib activity. In addition, we noted the presence of inflammatory cell infiltrate in the hematoxylin-eosin section of the responding area of GIST 6 (panels 2 and 3), of scattered lymphocytes in the hematoxylin-eosin section of the responding area of GIST 7 (panels 7 and 8), and of monocytes/macrophages in the hematoxylin-eosin section of the responding area of GIST 8 (panels 12 and 13). One SCGF-related signal was also detected by western blot in responding GISTs 14 and 16, but not in responding GIST 15 and non-responding GISTs 9-13 (Table 1 and Figure 5B).

SCGF is a secreted cytokine expressed in two distinct forms; SCGF-α is the full size form (323 amino acids, 35,695 Da), while SCGF-β is the shorter form (245 amino acids, 26,902 Da) characterized by a deletion within a conserved carbohydrate recognition domain [33]. These theoretical masses only partially explain the observed molecular weights in electrophoretic separations. The spectra from our MALDI-TOF-MS of tryptic peptides from two SCGF-positive bands (band 8 and 9 in Figure 1, lane E) were nearly identical in size and were attributed to the α form (lanes G and H in Figure 1A).

**Figure 6:**
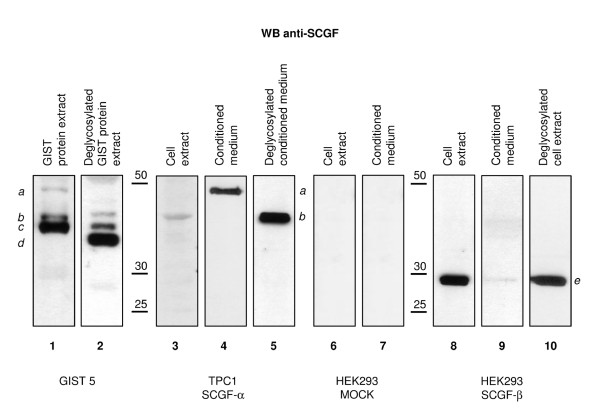
**Western blotting of SCGF after deglycosylation**. Western blotting with anti-SCGF-α/-β antibody. Lanes 1 and 2 compare SCGF patterns in the untreated (lane 1) and the deglycosylated (lane 2) protein extract of the GIST 5 sample. Lanes 3-5 contain a comparison of the cytoplasmatic (lane 3) and the secreted (lane 4) SCGF-α forms and the untreated (lane 4) and the deglycosylated secreted (lane 5) SCGF-α forms in the TPC1 cell line. Lanes 6 and 7 depict the expression of SCGF-α and -β in the cell extract and conditioned medium of a mock HEK293 cell line (negative control). Lanes 8-10 reflect SCGF-β expression in the cell extract (lane 8) and conditioned medium (lane 9) of the transfected HEK293 cell line; lanes 8 and 10 show the untreated and the deglycosylated SCGF-β forms in the transfected HEK293 cell line extract. The mature and secreted forms of SCGF-α detected in the GIST 5 sample (lane 1) and the TPC1 conditioned medium (lane 4) are indicated by *(a)*; *(b) *indicates an immature, cytoplasmatic, unglycosylated form of SCGF-α in the untreated (lane 1) and deglycosylated (lane 2) GIST 5 samples, and in the cell extract (lane 3) and conditioned medium of the TPC1 cell line after deglycosylation (lane 5); the form indicated by *(c) *was exclusively detected in the GIST 5 sample, was the quantitatively most relevant form in the untreated sample (lane 1), and was observed after deglycosylation (lane 2); *(d) *indicates the form exclusively detected and quantitatively most relevant in the deglycosylated GIST 5 sample (lane 2), corresponding to the protein backbone of SCGF-α (~36 kDa); *(e) *indicates the form detected in the untreated (lane 8) and deglycosylated (lane 10) cell extract of the transfected HEK293 cell line, corresponding to the primary structure of SCGF-β (~27 kDa).

Since SCGF has been described as O-glycosylated and sulphated, we deglycosylated the GIST 5 lysate to determine the sizes of SCGF bands before and after digestion. The immunoreactive band with the highest molecular weight (band *a *in Figure 6, lane 1) completely disappeared after deglycosylation; the intermediate band (band *b *in Figure 6, lane 1) remained unmodified, and the lowest band (band *c *in Figure 6, lane 1) exhibited an appreciable, apparently incomplete reduction in its molecular weight (band *d *in Figure 6, lane 2). Isoform *a *also occurred in conditioned medium (Figure 6, lane 4), as compared with isoforms expressed by the TPC1 cell line (Figure 6, lanes 3-5). Isoform *a *was equivalent to the fully glycosylated and secreted form, while the low-molecular weight isoform in the cell extract (band *b *in Figure 6, lanes 3 and 5) was equivalent to an immature, cytoplasmatic, unglycosylated form. Interestingly, we observed SCGF positivity for the isoforms corresponding to bands *a *and *b *in Figure 2, which were also observed in the conditioned medium (Figure 2, lane E) and in the cell extract (Figure 2, lane D) of the TPC1 cell line.

To further define the nature of SCGF isoforms expressed in the GIST 5 sample, we tested the possibility that the other form corresponded to isoform β. We transfected the HEK293 cell line that scored negative for SCGF proteins (Figure 6, lanes 6 and 7) with SCGF-β cDNA. Western blotting detected a protein with the size expected for the SCGF-β backbone (Figure 6, lanes 8-10) that did not correspond to band *d *of the GIST 5 sample (Figure 6, lane 2). Thus, we concluded that band *d *was not the SCGF-β form.

## Discussion

Few studies have correlated clinical response with histological response in GISTs after prolonged imatinib treatment. However, it is widely recognized that clinical outcome in stable or partially responsive GIST patients does not seem to be influenced by the duration of imatinib treatment, the histological response, or the size of the tumor [18]. In this study, we analyzed the proteomic, histological, immunohistochemical, and clinical features of a small group of GISTs resected after prolonged imatinib treatment.

SCGF, a novel cytokine, exerts its action on primitive hematopoietic progenitor cells. In combination with other hematopoietic growth factors such as granulocyte-macrophage colony-stimulating factor and erythropoietin, SCGF stimulates the formation of erythroid and granulocyte/macrophage colonies, although SCGF alone cannot induce colony formation [29]. Using a proteomic approach, we detected a large amount of SCGF in the protein extract of one imatinib-treated GIST sample (Figure 1). This sample also had a residual cell component of <10% and was negative or very slightly positive for CD117 staining. Biochemical analyses revealed the presence of a small number of KIT receptors with very low activation (data not shown). In parallel, we found extensive SCGF positivity in the abundant stromal component, which appeared homogenous, hypodense, and eosinophilic. This observation was replicated in part of the progressive lesion of another treated GIST case in which we identified strong SCGF positivity exclusively in CD117-negative areas. Interestingly, SCGF expression occurred in the imatinib-affected areas of three GISTs that was not detected in the unaffected or scarcely affected areas of the same tumors; SCGF-positive bands were also identified in two out three responding tumors but were absent in tumors from five non-responder patients.

A study carried out on *in vivo *material demonstrated that SCGF is strongly expressed in bone marrow and only faintly in lymphoid organs; in bone marrow, SCGF is concentrated in the cytoplasm of immature neutrophils, but not in myeloblasts, mature neutrophils, or the extracellular bone marrow fluid [34]. Since immature neutrophils play a role in tumor-induced immuno-inflammatory responses, SCGF may impact mechanisms regulating these responses. These observations are consistent with RNA expression patterns in mouse and human protein-encoding transcriptomes [35] and are attributed as follows: high expression levels to CD34+ cells, low expression levels to CD33+ (myeloid) cells, cardiomyocytes, and smooth muscle cells, and very low expression levels to all other cell lines or tissues.

Hiraoka [36] recently demonstrated that leukemia cell lines require self-secreted SCGF for their proliferation in tumors, indicating a putative autocrine SCGF mechanism, and that loop blockage with neutralizing antibody prevents extracellular SCGF from inducing apoptosis. Levina et al. [37] demonstrated that the high tumorigenic and metastatic potentials of lung cancer stem cells correlated with superior production of angiogenic factors and growth factors involved in cell proliferation and angiogenesis, describing increased levels of SCGF, stroma-derived factor 1α, and SCF in tumors from cancer stem cells in association with the stem cell phenotype. Gene-expression profiles from 35 childhood acute lymphoblastic leukemia matched diagnosis/relapse pairs, as well as 60 uniformly treated children at relapse, indicated that *SCGF *is significantly overexpressed at relapse [38].

The presence of SCGF in the CD117-negative stromal compartment of imatinib-treated GISTs suggests that its expression is associated with the histological response of GISTs to imatinib therapy. Our RT-PCR investigation revealed that SCGF is not actively transcribed in GIST samples, and thus it is difficult to determine the possible sources of SCGF in these specimens. A recent study described a subgroup of GISTs surgically resected after neoadjuvant imatinib treatment that exhibited reduced numbers of tumor cells in the hypocellular myxohyaline stroma, with small numbers of scattered atypical nuclei and occasional stromal hemorrhages [39]. A separate investigation assessed a GIST case treated with imatinib therapy for four weeks in which most of the tumor cells were replaced by myxoid stroma and the remaining tumor cells did not appear to be actively dividing [40]. However, to date no study has reported data on SCGF in GIST samples.

In our study, SCGF appeared as part of the stromal GIST component and, in particular, as part of the eosinophilic proteinaceous matrix described as myxoid, collagenous, or hyaline. Our proteomics experiment uncovered high plasma-protein content in treated tumors, and immunohistochemistry revealed the SCGF positivity in CD117-negative and CD68-positive areas. These observations could link SCGF positivity with the imatinib-induced inflammatory response that elicits monocyte/macrophage tissue migration, promoting scarring and removal of cell debris. CD68 positivity confirms macrophage infiltration, which may also explain the high level of C1q [41] in our GIST 5 proteomic analysis. Recent data support the hypothesis that induced type I maturation of dendritic cells is associated with a peak of SCGF production [1], supporting a pro-inflammatory role for this cytokine. It is therefore plausible that these are immunological reactions, and that liquidation of the dead tumor cells via macrophages leads to lesion shrinkage.

## Conclusions

SCGF function may be related to the imatinib-induced inflammation response in responding GIST patients. Further studies are necessary to identify the receptor of this cytokine, to further clarify its origin, and to determine the reason for its accumulation in some imatinib-treated GISTs. These investigations may answer fundamental questions about the composition of the stromal matrix after imatinib therapy and identify proteins related to desirable tumor response/behavior.

## Competing interests

The authors declare that they have no competing interests.

## Authors' contributions

LDV, FB, and IB planned the study and drafted the manuscript; FB, VH, AG, FM, ET, EF and MAP contributed to the design of the study and critically revised the manuscript; LDV, PM, FB, FM, Eva T, and EV performed western blotting and immunohistochemical experiments; SP contributed to data analysis. All the authors have read and approved the manuscript.

## Supplementary Material

Additional file 1 **Hematoxylin-eosin sections of representative slides from GIST 1, 3 and 4**. Click here for file

Additional file 2 **Espression of SCGF RNA in GIST samples 3, 4 and 5**. Click here for file
