# Supplementary figures and images for: Proteomic detection of a large amount of SCGFα in the stroma of GISTs after imatinib therapy
Source: J Transl Med. 2011 Sep 23;9:158. doi: 10.1186/1479-5876-9-158 (PMC3192683; doi:10.1186/1479-5876-9-158)

## Hematoxylin-eosin sections of representative slides from GIST 1, 3 and 4

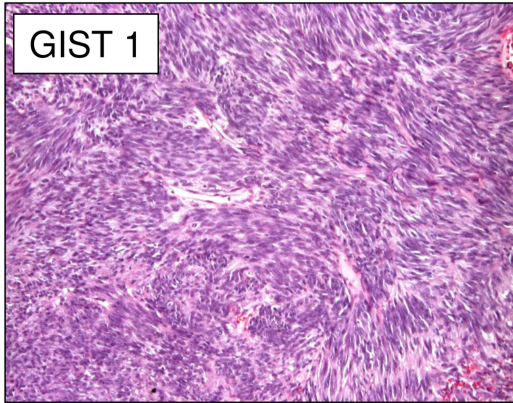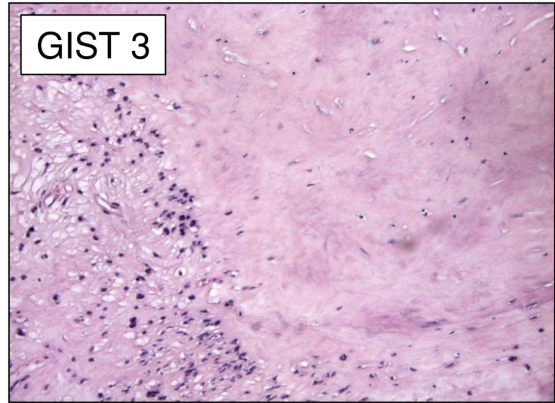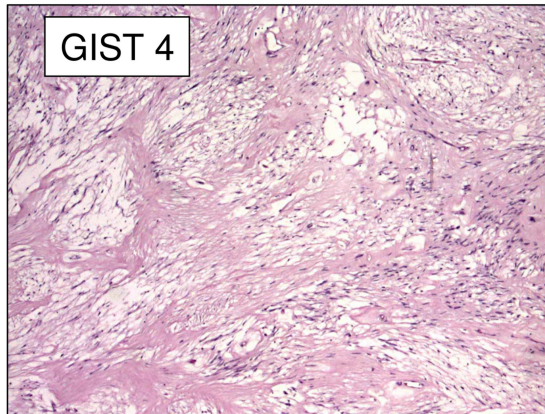

Supplement: Additional file 1 — Hematoxylin-eosin sections of representative slides from GIST 1, 3 and 4. [file 1479-5876-9-158-S1.PDF]
