# Supplementary material for: Proteomic detection of a large amount of SCGFα in the stroma of GISTs after imatinib therapy
Source: J Transl Med. 2011 Sep 23;9:158. doi: 10.1186/1479-5876-9-158 (PMC3192683; doi:10.1186/1479-5876-9-158)
Supplement: Additional file 2 — Espression of SCGF RNA in GIST samples 3, 4 and 5. [file 1479-5876-9-158-S2.PDF]

# Expression of SCGF RNA in GIST samples 3,4 and 5

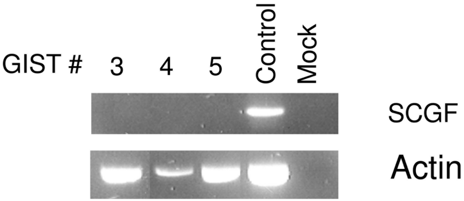


**Legend**

For reverse transcription (RT), 1 μg of purified RNA from GIST3, 4, and 5 samples and TPC1 cell line (positive control) was mixed with 1 μl deoxynucleotide triphosphate (dNTP) 10 mM, 1 μl oligo-dT 50 μM, H2O to 13 μl and incubated 10 min at 65°C. Then, 1 μl of Thermoscript reverse transcriptase, 4 μl of 5× first strand buffer, 1 μl of 0.1 mol/l DTT, 1 μl of RNaseOUT (40 U/μl) and sterile RNase-free water were added to a 20 μl total reaction volume. Reactions were incubated 60 min at 50°C and then 5 min at 85°C. In order to estimate the contamination in our reagents we produced a parallel RT-minus reaction where the RT-enzyme was substituted with water indicated as Mock. Reactions were incubated for 10 min at 25 °C, and the RNAs were then transcribed for 1 h at 55 °C. Subsequently, reverse transcriptase was inactivated at 85 °C for 5 min and RNA was degraded by digestion with 1 μl of RNase H (2 U/μl) at 37 °C for 20 min. and 1μl Thermoscript 25U/μl cDNAs were controlled by PCR amplification of β-actin.
PCR products (20 μl) were electrophoresed through a 2% agarose gel, which was subsequently stained with ethidium bromide to visualize the bands. Primers used to amplify SCGF were:  5’-CTGAAGCATCTGCAGGAA-3’ and 5’-TTCAGGCAGCCCTCCAAG-3’. Primers used to amplify actin were: 5’-CCGTGCTGCTGACCGAGGC-3’ and 5’-GGAAGGAAGGCTGGAAGAGT-3’.

**Additional file 2**
